# Isolation of cfDNA from spent culture media and its association with implantation rate and maternal immunomodulation

**DOI:** 10.1186/s13104-022-06151-8

**Published:** 2022-07-16

**Authors:** Amin Alizadegan, Maryam Akbarzadeh, Mohammad Sadegh Soltani-Zangbar, Roshanak Sambrani, Kobra Hamdi, Alieh Ghasemzadeh, Parvin Hakimi, Behnam Vahabzadeh, Hassan Dianat-Moghadam, Amir Mehdizadeh, Sina Mohammadinejad, Sanam Dolati, Sina Baharaghdam, Gholamreza Bayat, Mohammad Nouri, Mehdi Yousefi

**Affiliations:** 1 Department of Reproductive Biology, Faculty of Advanced Medical Sciences, Tabriz University of Medical Sciences, Tabriz, Iran; 2 Alzahra Hospital, Tabriz University of Medical Sciences, Tabriz, Iran; 3 Stem Cell Research Center, Tabriz University of Medical Science, Tabriz, Iran; 4 Woman’s Reproductive Health Research Center, Tabriz University of Medical Sciences, Tabriz, Iran; 5 Faculty of Veterinary and Paramedicine, Urmia Branch, Islamic Azad University, Urmia, Iran; 6 Department of Genetics and Molecular Biology, School of Medicine, Isfahan University of Medical Sciences, Isfahan, Iran; 7 Hematology and Oncology Research Center, Tabriz University of Medical Sciences, Tabriz, Iran; 8 Physical Medicine and Rehabilitation Research Center, Aging Research Institute, Tabriz University of Medical Sciences, Tabriz, Iran; 9 Department of Physiology-Pharmacology-Medical Physic, School of Medicine, Alborz University of Medical Sciences, Karaj, Iran; 10 Department of Immunology, School of Medicine, Tabriz University of Medical Sciences, Tabriz, Iran

**Keywords:** Cell-free DNA (cfDNA), Spent culture medium (SCM), IVF, PGT, And immune system

## Abstract

**Objectives:**

This investigation aims to evaluate the association between the concentration of cell-free DNA (cfDNA) in the spent culture medium (SCM) with implantation rate and the maternal immune system in the invitro fertilization (IVF). In this study, 30 embryos were cultured and scored according to Gardner's criteria. SCM was gathered on day five from every embryo to analyze the quantity of cfDNA. The real-time PCR technique evaluated the expression level of transcription factors, including Foxp3, RORγt, GATA3, and T-bet. The percentage of Th1, Th2, Th17, Treg, NK cells, and NK cells cytotoxicity was evaluated by flow cytometry.

**Results:**

The concentration of cfDNA in the β-HCG (-), β-HCG ( +), and ongoing pregnancy groups were 20.70 ± 9.224 ng/µL, 27.97 ± 7.990 ng/µL, and 28.91 ± 8.566 ng/µL, respectively. The ratio of Th1/Th2 and Th17/Treg reduced significantly in pregnant women, as well as the level of NK cells and NK cytotoxicity cells fell dramatically in the ongoing pregnancy group. The expression level of RORγt and T-bet declined while the expression level of Foxp3 and GATA3 increased considerably in pregnant mothers. Our investigation revealed that the concentration level of cfDNA in SCM could not be associated with implantation rate, prediction of ongoing pregnancy, and maternal immune system.

**Supplementary Information:**

The online version contains supplementary material available at 10.1186/s13104-022-06151-8.

## Introduction

Infertility is a problem in the worldwide that affect one in six couples in Western countries. According to the statistics, annually 60–80 million couples in the world suffer from infertility, various risk factors cause infertility, such as, the age of the woman, chromosomal abnormalities, ovulatory disorders, defective male fertility, hormonal disorders, immune response, cell phone use, sexual violence, stress, lifestyle related factors, including: smoking, obesity, alcohol, diet and chemical environment that reducing the chance of successful pregnancy [1–3].

Infertility severely affects the reproductive capabilities of mature partners, leading to increased application of assisted reproductive technology (ART) [4, 5]. The transmission of a single euploid embryo would decline the frequency of multiple pregnancy problems and miscarriage; while at the same time increases in vitro fertilization (IVF) accuracy and efficiency [6]. In this perspective, the laboratory uses numerous techniques to assess embryonic viability. These techniques comprise morphological evaluations based on distinct features noticed during embryonic development [7, 8], time-lapse imaging [9], metabolomics and proteomics assessment [10, 11], and micro-RNA analysis [12].

The cooperation of IVF technology and preimplantation genetic testing (PGT) has increased the chance of a healthy baby and normal pregnancy [13]. PGT can be performed by direct genetic evaluation of multiple cells or single cells that are removed from preimplantation embryos at the blastocyst stage (trophectoderm biopsy) or cleavage stage (blastomere biopsy), respectively [14, 15]. Although embryonic cell biopsy is still an invasive procedure, it has been extensively utilized in IVF units. Therefore, a non-invasive technique is required to detect embryos for aneuploidy or hereditary disorders. Lo, et al. [16] announced that embryonic cell-free DNA (cfDNA) exists in the peripheral blood of pregnant females, and cf-DNA can be applied for non-invasive PGT (niPGT). Researchers have reported that embryonic DNA can be found in blastocyst fluid and spent culture medium (SCM) [17, 18]. It seems that the existence of cf-DNA in the blastocoel cavity is due to natural apoptosis during embryonic development [19]. Shamonki et al. [20] stated that the niPGT results of cfDNA in SCM were compatible with trophectoderm (TE) biopsy. Other research also revealed that aneuploid niPGT (niPGT-A) from SCM was less susceptible to embryonic mosaicism mistakes, and niPGT-A was more reliable than PGT-A from TE biopsy [21].

A healthy pregnancy requires intricate interactions between the decidual immune cells of the mother and the fetal trophoblasts, which promote the growth and development of the embryo in the uterus. At the same time, the immunological mechanism of the mother stays mainly intact. Macrophages, DCs, T cells, and NK cells play a vital role in modulating the uterine environment to ensure a healthy pregnancy [22]. macrophages play an important role in pregnancy, in early pregnancy macrophage involved in trophoblast invasion, tissue, vascular remodeling, and tolerance to the semiallogeneic fetus, macrophages provide a significant contribute to fertilization, implantation and decidualization and Their dysfunction is implicated in pregnancy disorders, like infertility, intrauterine growth restriction, preeclampsia, recurrent spontaneous abortion, and preterm labor [23]. at the same time, T cells exerts an essential role in promoting maternal–fetal tolerance, Embryo implantation, Placenta development and Fetus survival by controlling the trophoblast invasion and angiogenesis [24, 25]. Moreover, NK cells are the most abundant immune cells of decidua in first trimester of gestation and affect pregnancy outcome by secreting cytokines, chemokines, and angiogenic mediators, among immune cells, NK cells Make significant contributions to spiral artery remodeling, trophoblast invasion, response to pathogens, and also decidualization, which have an important role in outcome of pregnancy [26] The predominant frequency of NK cells in the blood circulation is CD16^+^CD56^dim^ NK cells, whereas the majority of NK cells in the endometrium are CD16^−^CD56^bright^ NK cells [27]. A recent study showed that the percentage of CD16 + CD56dim NK cells increases in patients who experienced recurrent pregnancy loss [28]. During pregnancy, T lymphocytes play a critical function in immunostimulation and immunoregulation [29]. Investigations have shown that during a healthy pregnancy, the percentage of Th2 outnumbers Th1, which can protect the fetus from the maternal immune system [30, 31]. The recent paradigm for healthy pregnancy has been extended to Th1/Th2 and Th17/regulatory T (Treg) [32, 33]. This carefully controlled process between defense and tolerance includes a unique kinetic profile for Treg cells located at the maternal–fetal interface [34]. This research aims to evaluate the association between the concentration of cfDNA in SCM with implantation rate and the maternal immune system in IVF cycles.

## Main text

### Materials and methods

#### Study design

Intracytoplasmic sperm injection (ICSI) was utilized to fertilize oocytes. Thirty embryos were cultured based on the conventional blastocyst culture process. SCM was gathered on day five from every embryo to analyze the quantity of cfDNA. Additionally, five media drops were retained in the same conditions without interaction with embryos to serve as controls. The quantification of cfDNA was assessed by Nanodrop Spectrophotometer. Blastocysts morphology was also scored According to blastocyst scoring system (Gardner's criteria) [35], that is, Grade 1: early blastocyst, wherein the blastocele is less than half the volume of the embryo; Grade 2: blastocyst, wherein the blastocele is greater than or equal to half of the volume of the embryo; Grade 3: full blastocyst, wherein the blastocele completely fills the embryo; Grade 4: expanded blastocyst, wherein the blastocele volume is larger than that of the early embryo and the zona pellucida is thinning; Grade 5: hatching blastocyst, which the trophectoderm has started to herniate through the zona pellucida; and Grade 6: hatched blastocyst, in which the blastocyst has completely escaped from the zona pellucida. The development of the inner cell mass (ICM) and trophectoderm was also assessed. The ICM grading was as follows: A: many cells that are tightly packed; B: several cells that are loosely grouped; or C: very few cells. The trophectoderm grading was as follows: A: many cells forming a tightly knit epithelium; B: a few cells; or C: very few cells forming a loose epithelium [36]. Finally, single embryo was transferred to each patient.

#### Maternal blood sampling

Blood samples were obtained from 30 women two weeks after IVF–embryo transfer. Blood samples were used for β-human chorionic gonadotropin (β-HCG) and immunological factors analysis. Among the β-HCG ( +) group, blood samples were taken from pregnant women at 12 weeks of gestation for further immunological factors examination. Briefly, 10 ml of blood were taken in the heparinized tube from participants under aseptic condition. PBMCs were isolated from freshly drawn blood by 1.077 g/ml Ficoll density-gradient centrifugation. Please, reformulate this sentence in a more fluent way [37]. Separated PBMCs were applied for gene expression and lymphocyte percentage analysis.

#### Real time PCR

Isolated PBMCs were incubated with RNX-PLUS Solution to extract total RNA (Sina Clon, Iran). Revert Aid Reverse Transcriptase kit was used according to manufacture instruction to synthesis Complementary DNA (cDNA) (Thermo Fisher, MA). The real-time PCR technique evaluated the expression level of transcription factors associated with the maternal immune system, including Foxp3, RORγt, GATA3, and T-bet.

#### Flow cytometry

The percentage of Th1, Th2, Th17, Treg, NK cells, and NK cells cytotoxicity in isolated PBMCs from 30 participants was evaluated by flow cytometry. For Th1 and Th2 cells detection in PBMCs, cells were dyed with FITC-labeled anti CD4, PE-labeled anti-IL-4 (for Th1) and APC-labeled anti-IFN-γ (for Th2) (BD Biosciences, CA, USA) for 30 min at room temperatur. PBMCs were stained by FITC-conjugated anti-CD4 antibodies at 4 °C for 15 min and after the washing process cells were incubated with anti-IL-17-APC antibodies for 20 min at room temperature (BD Biosciences, CA, USA) for Th17 recognition. PBMCs were incubated for 45 min at 4 °C with FITC-conjugated anti-CD4, and anti-CD127-APC, and anti-CD25-PE-conjugated antibodies (BD Biosciences, CA, USA) for Treg detection, and anti-CD56 fluorochrome-conjugated antibody was used for NK cells frequency assessment. PBMCs were stained and incubated with antibodies based on previous work [38, 39]. The flow cytometry-based method was assessed NK cells cytotoxicity [40]. All samples were analyzed on the same day using a FACS via flow cytometer (BD Biosciences, CA, USA).

#### Statistical analysis

Data were displayed by Mean ± SD. Statistical analysis was performed by SPSS (Ver. 24.0). Kruskal–Wallis one-way analysis was performed to assess statistical significance between groups. Graph Pad Prism (Ver. 8.00) was used to illustrate all graphs. P-values lower than 0.05 was presumed as statistically significant.

## Results

### Embryo grade and pregnancy

β-HCG of 30 participants undergoing IVF–embryo transfer was tested. 18 and 12 women were negative and positive for the β-HCG test, respectively. Among participants with the positive β-HCG test, 9 women exhibited ongoing pregnancy for the first trimesters. The morphology of embryos was graded according to Gardner’s criteria (Table 1).

**Table 1:** embryo grade in β-HCG-, β-HCG + groups

	Embryo Grade					
	1AA	2AA	3AA	4AA	4BB	4CC
β-HCG-(N = 18)	5	3	2	2	3	**3**
β-HCG + (N = 12)	2	3	2	1	2	**2**
Firs trimesters(N = 9)	2	2	1	1	2	**1**

### cfDNA concentration

**Fig. 1:**
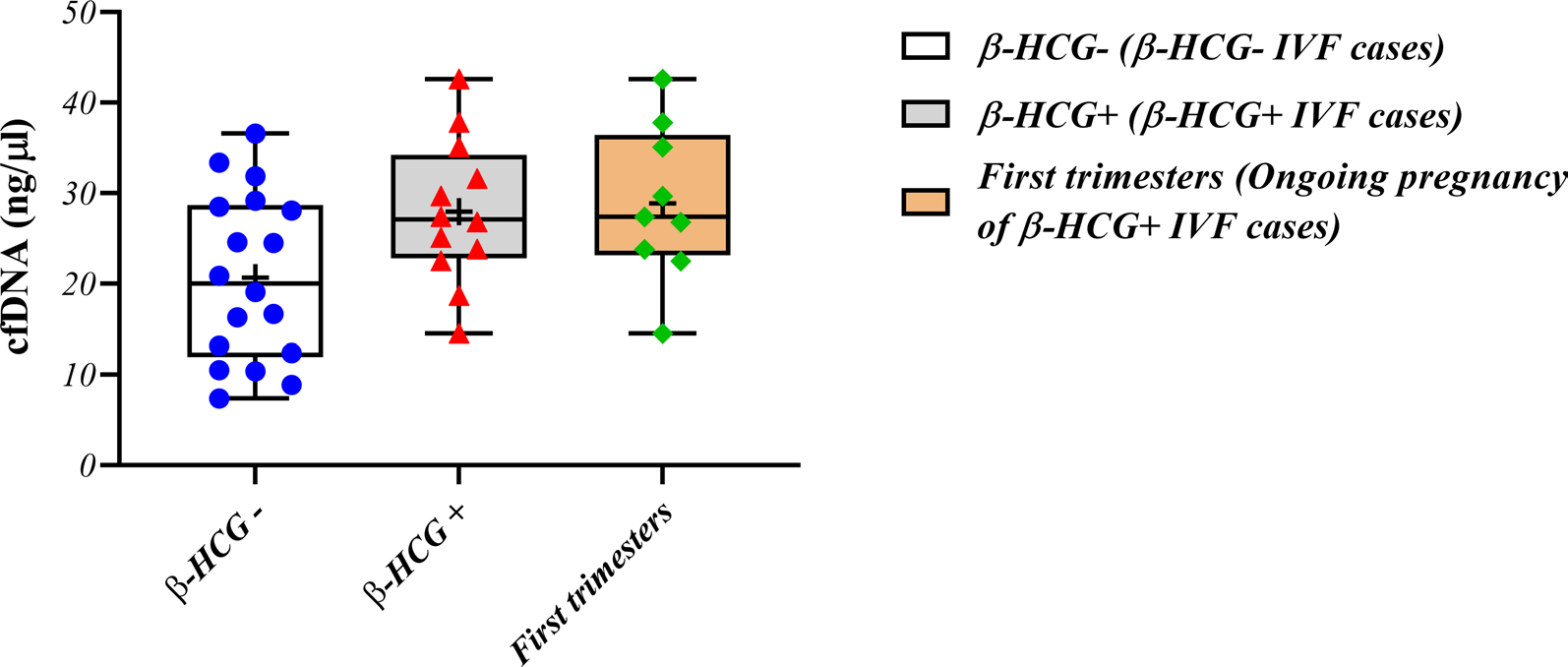
Concentration of cfDNA (ng/µL) in the in the β-HCG (-), β-HCG ( +) and ongoing pregnancy groups. Data are presented as mean ± standard division

The concentration of cfDNA in SCM was measured by Nanodrop. 5 samples were assessed as a control to check media contamination and the amount of DNA in these media was negligible (0.9000 ± 0.9798 ng/µL). The cfDNA concentration in β-HCG (-), β-HCG ( +), and ongoing pregnancy groups were 20.70 ± 9.224 ng/µL, 27.97 ± 7.990 ng/µL, and 28.91 ± 8.566 ng/µL, respectively. The P-value between β-HCG (-) and β-HCG ( +) was 0.1775. The P-values for β-HCG (-) and β-HCG ( +) compared to ongoing pregnancy were 0.1548 and > 0.9999, respectively (Fig. 1 and Additional file 1: Table S1).

### Flow cytometry analysis

**Fig. 2:**
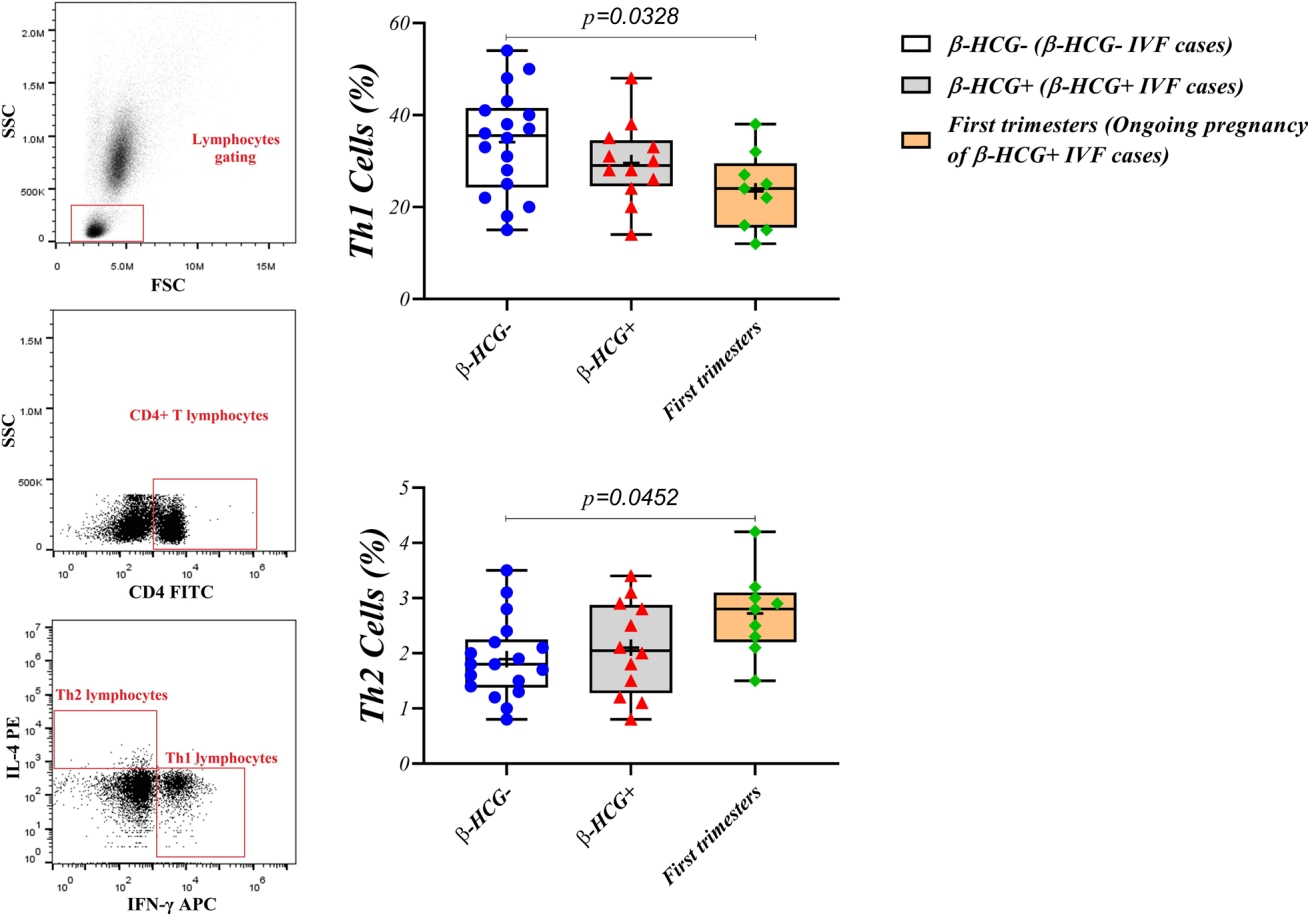
Frequency of Th1 and Th2 in the β-HCG (-), β-HCG ( +) and ongoing pregnancy groups. Data are presented as mean ± standard division. P < 0.05 was considered as statistically significant

The flow cytometry results are displayed in Additional file 2: Table S2. The results indicated significantly declined Th1 level in pregnant women compared to the β-HCG (-) group (23.44 ± 8.368 versus 34.11 ± 11.20, p = 0.0328, respectively). While, the frequency of Th2 increased considerably in ongoing pregnancy group in comparison with β-HCG (-) (2.722 ± 0.7612 versus 1.894 ± 0.7125, p = 0.0452, respectively) (Fig. 2 and Additional file 2: Table S2). Additionally, the percentage of Th17 decreased in the first trimester women compared to both β-HCG (-) and β-HCG ( +) groups (1.911 ± 1.135 versus 3.528 ± 1.554 and 3.383 ± 1.407, p = 0.0171 and p = 0.0454, respectively. In contrast, Treg frequency increased noticeably in ongoing pregnancy group compared to both β-HCG (-) and β-HCG ( +) groups (8.167 ± 3.689 versus 4.094 ± 1.621 and 4.300 ± 2.022, p = 0.0286 and p = 0.0422, respectively) (Additional file 3: Figure S1 and Additional file 2: Table S2). Besides, Th1/Th2 (8.711 ± 2.302 versus 19.26 ± 7.421 and 15.43 ± 5.295, p < 0.0001 and p = 0.0036, respectively) and Th17/Treg (0.3100 ± 0.2226 versus 0.9189 ± 0.3455 and 0.8450 ± 0.2983, p < 0.0001 and p = 0.0005, respectively) ratios significantly reduced in pregnant women compared to β-HCG (-) and β-HCG ( +) (Additional file 4: Figure S2 and Additional file 2: Table S2).

Regarding the NK cells, flow cytometry results showed significant reduction in NK cells number (7.111 ± 2.369 versus 12.82 ± 4.127 and 11.17 ± 3.129, p = 0.0005 and p = 0.0093, respectively) and cytotoxicity (7.889 ± 2.759 versus 14.59 ± 4.678 and 12.17 ± 3.433, p = 0.0048 and p = 0.0450, respectively) in the ongoing pregnancy group compared to both previously mentioned groups (Additional file 5: Figure S3 A/B and Additional file 2: Table S2).

### Gene expression analysis

The real-time PCR technique was exerted to evaluate the expression level of transcription factors such as Foxp3, RORγt, GATA3, and T-bet (Additional file 2: Table S2). The results exhibited that the expression level of RORγt (0.3333 ± 0.1732 versus 1.000 ± 0.07096 and 0.8000 ± 0.5187, p < 0.0001 and p = 0.0330, respectively) and T-bet (0.6444 ± 0.3504 versus 1.000 ± 0.1033 and 0.8583 ± 0.2906, p = 0.0430 and p > 0.05) declined in pregnant women compared to β-HCG (-) and β-HCG ( +) groups. On the other side, the expression level of Foxp3 (1.767 ± 0.3391 versus 1.000 ± 0.1328 and 1.208 ± 0.5900, p = 0.0003 and p = 0.0396, respectively) and GATA3 (1.767 ± 0.6856 versus 1.000 ± 0.09165 and 1.158 ± 0.3059, p = 0.0285 and p > 0.05, respectively) considerably increased in participants that experienced the first trimesters of pregnancy in comparison with the β-HCG (-) and β-HCG ( +) groups (Additional file 6: Figure S4 and Additional file 2: Table S2).

## Discussion

In clinical experience, some embryos with excellent morphology lead to failed pregnancy, even spontaneous termination after transfer. These might be due to a chromosomal inversion, deletion, translocation, or DNA mutation. Thus, DNA analysis is required before embryo implantation [41]. Currently, TE biopsy has been used for chromosomal screening before embryo implantation and this technique is vastly reproducible between ART clinics [42]. Although, the origin of cfDNA is unknown, embryonic cells apoptosis may contribute to the presence of cfDNA [43, 44]. Therefore, cfDNA in the SCM could be derived from each cell line and represents the entire circumstance of blastocysts more accurately than TE biopsy [45]. Although, S. Stigliani et al. [46] found that the quantity of DNA in embryos with poor quality cleavage is more than high-scored embryos and the ratio of mitochondrial DNA/genomic DNA in SCM was related to good implantation results, we could not find any significant association between the amounts of total cfDNA in SCM and the rate of implantation in our study. These differences might be due to the appliance of various techniques for assessing cfDNA in SCM. The recently published research showed pieces of evidence that the well-scored embryos with excellent morphological characteristics have a high amount of cfDNA in blastocoel fluid [19]. These findings strengthen the notion that DNA molecules are released into SCM from embryonic cells as a method for correcting aneuploidies [47]. Some researchers have also believed that the presence of cfDNA in embryonic culture is produced by apoptosis during embryonic physiological development [48, 49]. Even though we observed that the quantity of cfDNA was slightly higher in the pregnant groups; however, the difference was not statically significant.

Many investigations have found that whereas Th1 cells are prominent in abortion, the presence of Th2 cells has also been observed in recurrent pregnancy loss [50, 51]. However, the Th17/Treg balance is disturbed in particular pregnancy disorders such as preterm birth, preeclampsia, and URPL [38, 52]. In concordance with previous studies, our results showed that the Th1/Th2 and Th17/Treg ratios considerably declined in ongoing pregnancy group. Gene expression analysis also confirmed that the transcription factors involved in Th17 (RORγt) and Th1 (T-bet) development decreased in pregnant women. While, the expression level of FOXP3 and GATA3 significantly increased in pregnant women, which are crucial for Treg and Th1 development, respectively. Patients that suffer from recurrent pregnancy loss had a high proportion of peripheral blood NK cells; while, the percentage of NK cells in the endometrium is significantly low [28]. We also demonstrated that the frequency and activity of NK cells declined in peripheral blood of the group which experienced the first trimester of pregnancy.

## Conclusion

In conclusion, our investigation revealed that cfDNA concentration level in spent culture medium could not be associated with implantation rate, prediction of ongoing pregnancy, and maternal immune system. Due to increased need for regular genetic testing before the implantation process, PGT has recently been widely used in ART. While, researches have proven the existence of significant cfDNA in embryonic SCM, numerous technological hurdles must be overcome before NI-PGT can be considered as a credible source of embryonic genetic information. Comprehensive investigations are necessary to identify how accurately cfDNA indicates the genetic composition of the entire embryo and assess the medical effectiveness of these techniques on pregnancy outcomes.

## Limitations

A limitation of our study was small sample size.

## Supplementary Information

**Additional file 1: Table S1.** cfDNA content in spent culture media of groups.

**Additional file 2: Table S2.** Immunological features of IVF implanted women in different situation.

**Additional file 3: Figure S1.** The frequency of Th17 and Treg in the β-HCG (-), β-HCG ( +) and ongoing pregnancy groups. Data are presented as mean ± standard division. P < 0.05 was considered as statistically significant.

**Additional file 4: Figure S2.** The ratio of Th1/Th2 and Th17/Treg in the β-HCG (-), β-HCG ( +) and ongoing pregnancy groups. Data are presented as mean ± standard division. P < 0.05 was considered as statistically significant.

**Additional file 5: Figure S3.** A) The percentage of NK cells and B) the percentage of NK cell cytotoxicity in the β-HCG (-), β-HCG ( +) and ongoing pregnancy groups. Data are presented as mean ± standard division. P < 0.05 was considered as statistically significant.

**Additional file 6: Figure S4.** The expression level of transcription factors involved in the maternal immune system. Data are presented as mean ± standard division. P < 0.05 was considered as statistically significant.

## Data Availability

All the necessary data are presented herewith. However if needed, raw data on excel format can be availed on reasonable request from the corresponding author.

## Abbreviations

ART: Assisted reproductive technology
β-HCG: β-Human chorionic gonadotropin
cfDNA: Cell-free DNA
DCs: Dendritic cells
Foxp3: Forkhead box P3
ICM: Inner cell mass
ICSI: Intracytoplasmic sperm injection
IVF: In-vitro fertilization
niPGT: Non-invasive PGT
PBMCs: Peripheral blood mononuclear cells
PGT: Preimplantation genetic testing
RORγt: Receptor-related orphan receptor γt
SCM: Spent culture medium
TE: Trophectoderm
URPL: Unexplained recurrent pregnancy loss
