# Supplementary material for: Isolation of cfDNA from spent culture media and its association with implantation rate and maternal immunomodulation
Source: BMC Res Notes. 2022 Jul 16;15:259. doi: 10.1186/s13104-022-06151-8 (PMC9288726; doi:10.1186/s13104-022-06151-8)
Supplement: Supplementary file 1 — Additional file 1: Table S1. cfDNA content in spent culture media of groups. [file 13104_2022_6151_MOESM1_ESM.docx]

Table S1: cfDNA content in spent culture media of groups.

|  | β-HCG- (N=18)  mean±SD | β-HCG+ (N=12)  mean±SD | First trimester (N=9)  mean±SD |  | p Value |  |
| --- | --- | --- | --- | --- | --- | --- |
|  |  |  |  | β-HCG- vs β-HCG+ | β-HCG- vs Ongoing pregnancy | β-HCG+ vs Ongoing pregnancy |
| cfDNA (ng/μL) | 20.70±9.224 | 27.97±7.990 | 28.91±8.566 | **0.1775** | **0.1548** | **>0.9999** |
