# Supplementary material for: Isolation of cfDNA from spent culture media and its association with implantation rate and maternal immunomodulation
Source: BMC Res Notes. 2022 Jul 16;15:259. doi: 10.1186/s13104-022-06151-8 (PMC9288726; doi:10.1186/s13104-022-06151-8)
Supplement: Supplementary file 2 — Additional file 2: Table S2. Immunological features of IVF implanted women in different situation. [file 13104_2022_6151_MOESM2_ESM.docx]

Table S2: Immunological features of IVF implanted women in different situation.

| Subject | β-HCG-  mean±SD  (N=18) | β-HCG+  mean±SD  (N=12) | First trimesters  mean±SD  (N=9) | p Value | | |
| --- | --- | --- | --- | --- | --- | --- |
|  |  |  |  | **β-HCG- vs β-HCG+** | **β-HCG- vs First trimesters** | **β-HCG+ vs First trimesters** |
| Flow cytometry | | | | | | |
| Th1 (%) | 34.11±11.20 | 29.58±8.723 | 23.44±8.368 | NS | 0.0328 | NS |
| Th2 (%) | 1.894±0.7125 | 2.100±0.8496 | 2.722±0.7612 | NS | 0.0452 | NS |
| Th1/Th2 | 19.26±7.421 | 15.43±5.295 | 8.711±2.302 | NS | <0.0001 | 0.0036 |
| Th17 (%) | 3.528±1.554 | 3.383±1.407 | 1.911±1.135 | NS | 0.0171 | 0.0454 |
| Treg (%) | 4.094±1.621 | 4.300±2.022 | 8.167±3.689 | NS | 0.0286 | 0.0422 |
| Th17/Treg | 0.9189±0.3455 | 0.8450±0.2983 | 0.3100±0.2226 | NS | <0.0001 | 0.0005 |
| NK (%) | 12.82±4.127 | 11.17±3.129 | 7.111±2.369 | NS | 0.0005 | 0.0093 |
| NK cells cytotoxicity (%) | 14.59±4.678 | 12.17±3.433 | 7.889±2.759 | NS | 0.0048 | 0.0450 |
| Relative Gene Expression (Fold Change) | | | | | | |
| *T-bet* | 1.000±0.1033 | 0.8583±0.2906 | 0.6444±0.3504 | NS | 0.0430 | NS |
| *GATA-3* | 1.000±0.09165 | 1.158±0.3059 | 1.767±0.6856 | NS | 0.0285 | NS |
| *RORγT* | 1.000±0.07096 | 0.8000±0.5187 | 0.3333±0.1732 | NS | <0.0001 | 0.0330 |
| *FoxP-3* | 1.000±0.1328 | 1.208±0.5900 | 1.767±0.3391 | NS | 0.0003 | 0.0396 |
