# Supplementary figures and images for: Isolation of cfDNA from spent culture media and its association with implantation rate and maternal immunomodulation
Source: BMC Res Notes. 2022 Jul 16;15:259. doi: 10.1186/s13104-022-06151-8 (PMC9288726; doi:10.1186/s13104-022-06151-8)

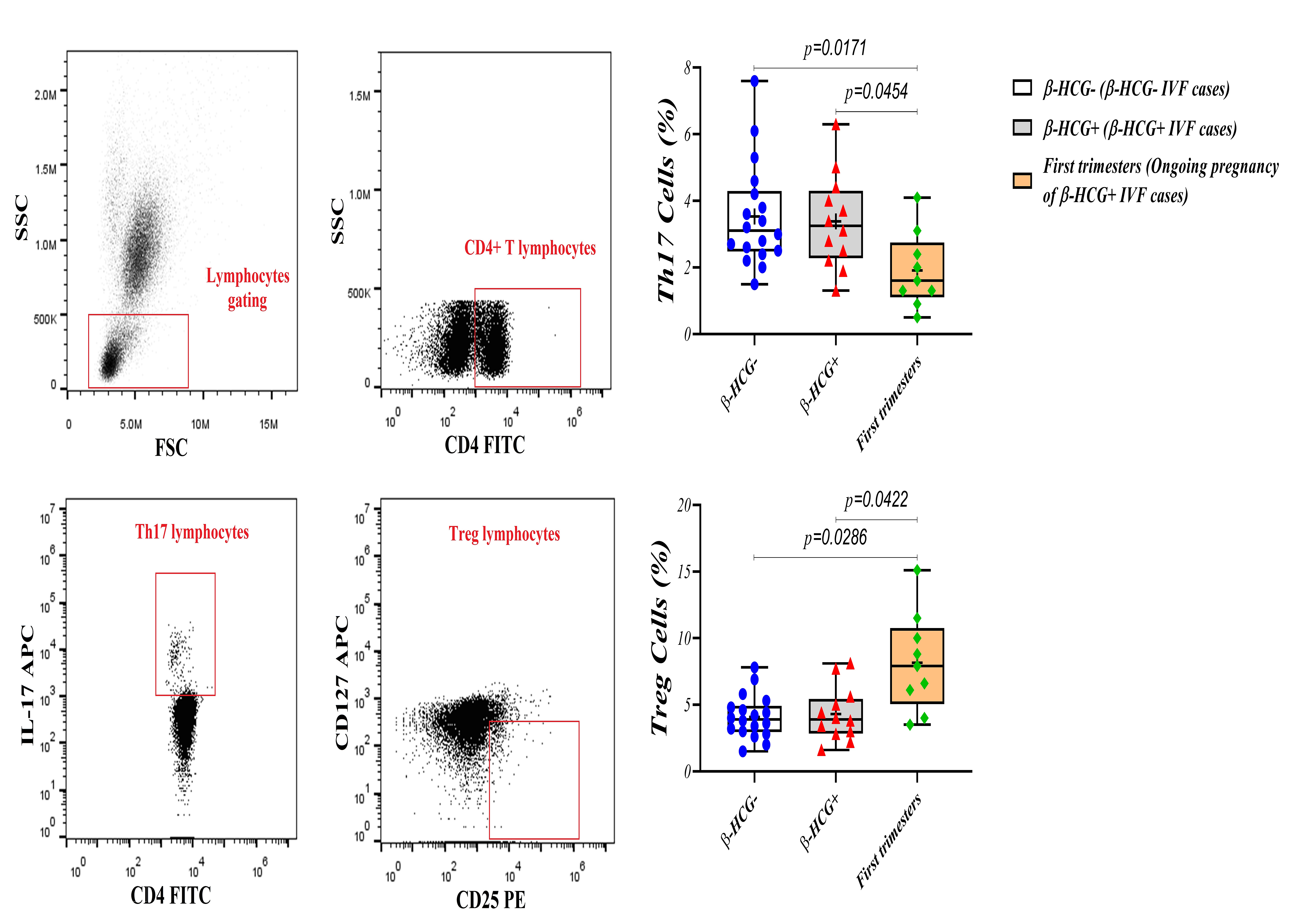

Supplement: Supplementary file 3 — Additional file 3: Figure S1. The frequency of Th17 and Treg in the β-HCG (-), β-HCG ( +) and ongoing pregnancy groups. Data are presented as mean ± standard division. P < 0.05 was considered as statistically significant. [file 13104_2022_6151_MOESM3_ESM.tiff]

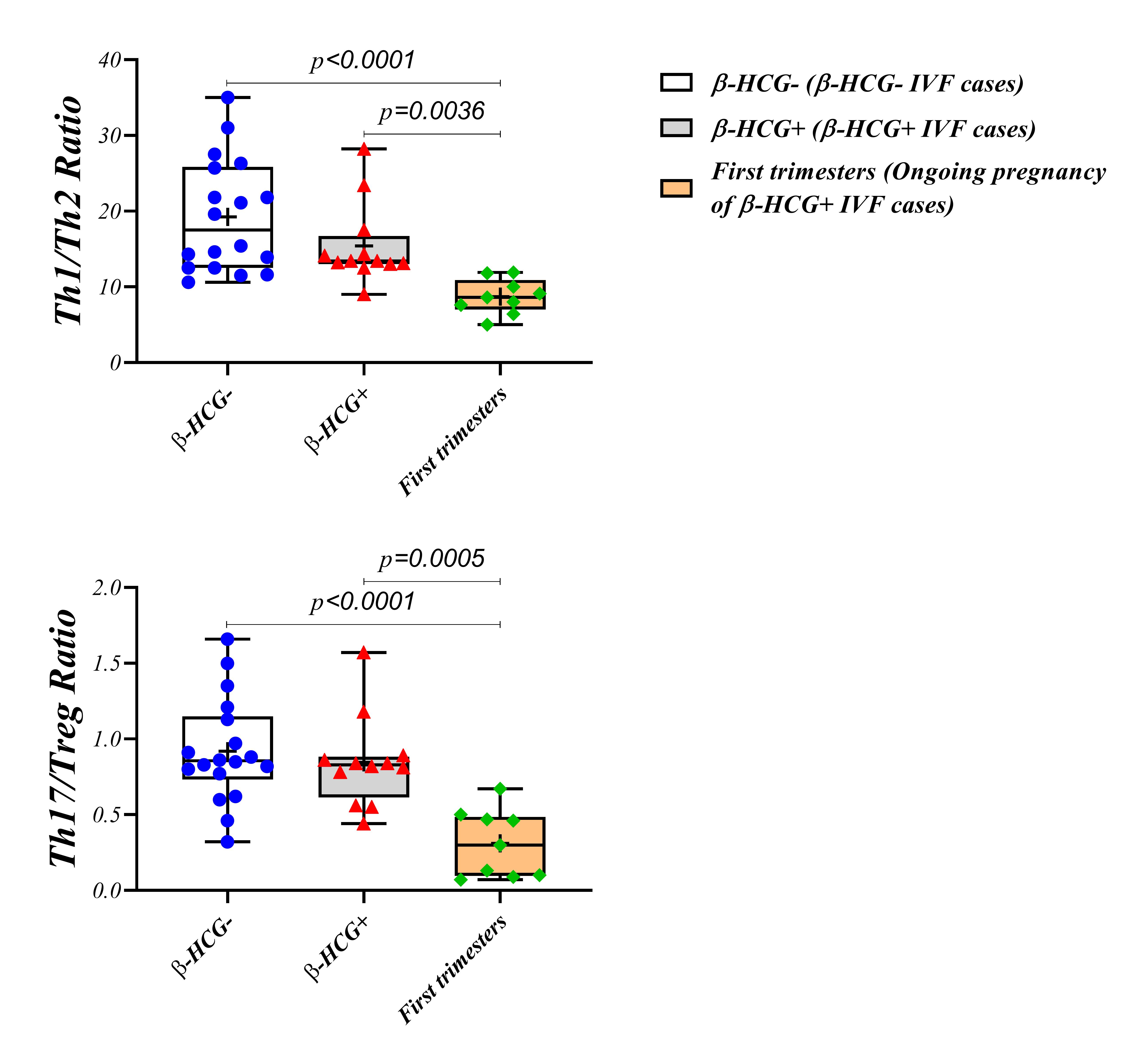

Supplement: Supplementary file 4 — Additional file 4: Figure S2. The ratio of Th1/Th2 and Th17/Treg in the β-HCG (-), β-HCG ( +) and ongoing pregnancy groups. Data are presented as mean ± standard division. P < 0.05 was considered as statistically significant. [file 13104_2022_6151_MOESM4_ESM.tiff]

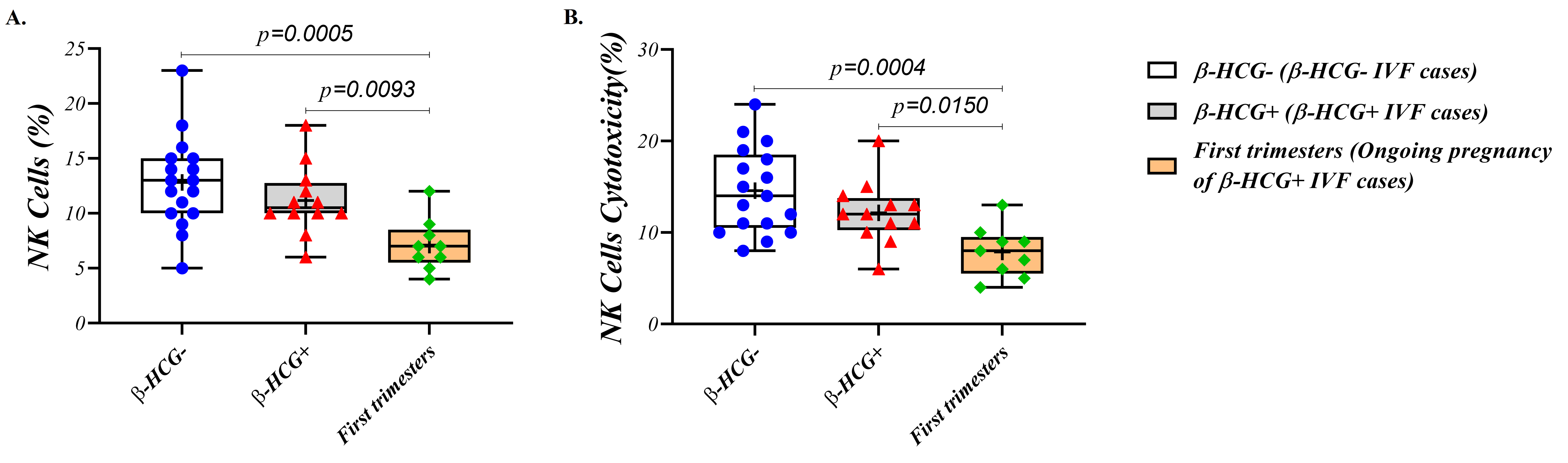

Supplement: Supplementary file 5 — Additional file 5: Figure S3. A) The percentage of NK cells and B) the percentage of NK cell cytotoxicity in the β-HCG (-), β-HCG ( +) and ongoing pregnancy groups. Data are presented as mean ± standard division. P < 0.05 was considered as statistically significant. [file 13104_2022_6151_MOESM5_ESM.tif]

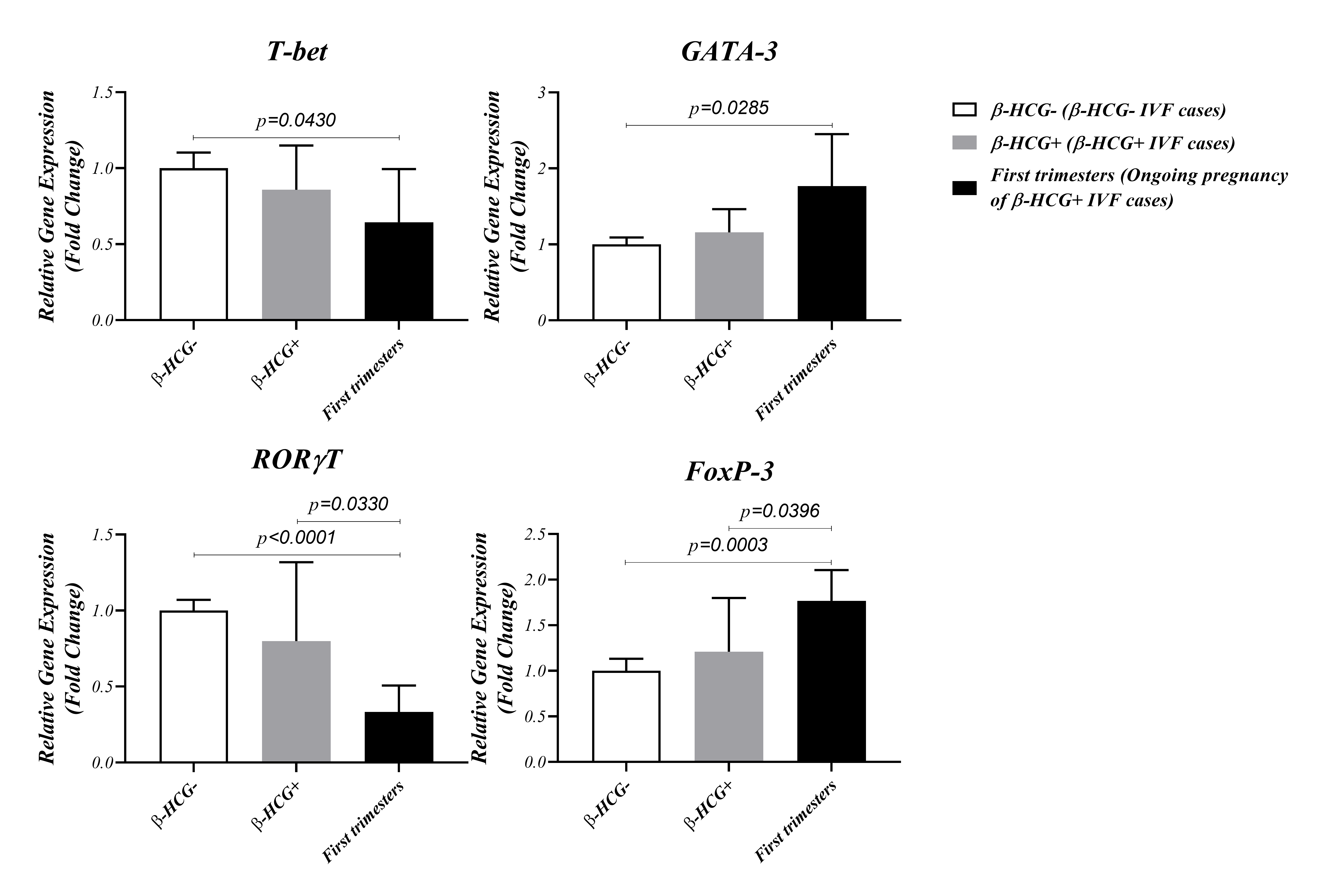

Supplement: Supplementary file 6 — Additional file 6: Figure S4. The expression level of transcription factors involved in the maternal immune system. Data are presented as mean ± standard division. P < 0.05 was considered as statistically significant. [file 13104_2022_6151_MOESM6_ESM.tiff]
